# A one-stage anchor-free keypoints detection model for fast electric vehicle charging port detection and pose extraction

**DOI:** 10.1038/s41598-025-98203-9

**Published:** 2025-05-17

**Authors:** Feifei Hou, Qiwen Meng, Xinyu Fan, Yijun Wang

**Affiliations:** School of Automation, Central South University, Changsha, PR China

**Keywords:** Electric vehicle (EV), Charging Port, Keypoints detection, Perspective-n-point (PnP), Deep learning (DL), Computer science, Information technology

## Abstract

As intelligent technologies advance in electric vehicles (EVs), automatic unmanned charging systems are becoming increasingly prevalent. A key breakthrough lies in developing efficient methods to identify and locate charging ports. However, challenges such as high sensor costs, compromised robustness in complex environments, and stringent computational demands remain. To address these issues, this study introduces FasterEVPoints, a state-of-the-art convolutional neural network (CNN) model integrating partial convolution (PConv) with FasterNet. Tailored to pinpoint critical points of EV charging ports, FasterEVPoints incorporates the perspective-n-point (PnP) algorithm for pose extraction and the bundle adjustment (BA) optimization algorithm for refined pose accuracy. This approach operates effectively with only a single RGB camera, ensuring precise localization with minimal hardware. Experiments demonstrate that in complex lighting scenarios, FasterEVPoints boasts 95% detection accuracy on a proprietary dataset with a positioning error of less than 2 cm at a 50 cm distance. Furthermore, when integrated into the you only look once X (YOLOX) framework with parameters comparable to YOLOX-Tiny, FasterEVPoints delivers similar accuracy while consuming only 73% of the computational load and 66% of the parameters compared to YOLOX-Tiny. This exceptional efficiency, combined with high detection accuracy, establishes FasterEVPoints as a practical and scalable solution for real-world autonomous EV charging applications.

## Introduction

With the rapid growth of new energy markets, automatic charging mechanisms have become indispensable for enabling unmanned and intelligent operation of electric vehicles (EVs)^1,2^. This technology facilitates a seamless and efficient charging process, significantly enhancing the user experience^3^. Accurate and robust identification of the charging port’s position and orientation is paramount, forming the foundation for the development and deployment of automatic charging systems^4^. This capability ensures that EVs can be charged without human intervention, thereby improving both the convenience and efficiency of EV usage. Furthermore, it enables the creation of advanced systems capable of automatically aligning charging equipment with the vehicle’s charging interface, driving further advancements in EV charging automation^5^. Ultimately, automatic charging mechanisms play a pivotal role in the future of EV technology, accelerating its widespread adoption and enhancing its overall functionality.

In recent years, researchers have explored a wide range of methods to tackle the challenge of charging port detection and localization, spanning from traditional image processing techniques^6–9^ to deep learning -based approaches^10–12^. Early research primarily focused on leveraging conventional image processing techniques for this task. For instance, Zhang^13^ proposed a two-stage charging port detection and localization algorithm utilizing the HSI color model. While this method achieved satisfactory localization accuracy, it exhibited limited real-time performance, requiring approximately 1051.2 ms per frame for processing. Similarly, contour-based methods^7,14^ have also been studied; however, their effectiveness remains constrained in complex environments. Despite these advancements, the development of more efficient and robust solutions remains imperative to address the challenges associated with charging port detection and localization in real-world scenarios.

Alternative approaches have explored the utilization of diverse sensors for charging port recognition and localization. Pérez et al.^6^ employed an infrared system, incorporating infrared LED markers and a camera to precisely determine the charging port’s position. Tadic^8^ applied depth camera localization techniques for EV charging port localization, while Miseikis et al.^15^ introduced a stereo camera-based method to guide the plugging and unplugging of chargers. However, this approach relies heavily on intricate image processing pipelines and is highly dependent on controlled lighting conditions and model accuracy. Although these methods offer improved localization accuracy, they introduce significant complexities, increase costs, and may limit generalization in dynamic and unstructured environments. These approaches typically leverage multiple sensors to acquire data, utilizing either traditional image processing techniques or deep neural networks for charging port identification and localization.

Recent research has made significant strides in object detection and recognition under complex environments^16–18^. Zhang et al.^19^ proposed a densely cropped and local attention object detector network (DCLANet), which integrates an attention mechanism into YOLOv5 and employs a non-uniform image cropping strategy to enhance small-object detection in low-altitude aerial images. This approach has demonstrated remarkable improvements in detecting tiny and nonrigid objects under varying environmental conditions. A transformer-based person detection network (VTSaRNet)^20^ was introduced, leveraging bimodal RGB-T data and transformer-based fusion techniques to improve detection robustness under challenging illumination conditions. This model effectively combines visible and thermal information to enhance detection performance in search and rescue (SaR) applications. Additionally, a lightweight distillation network (APDNet)^21^ was developed specifically for aerial person detection on edge devices. APDNet utilizes knowledge distillation and quantization-aware training to achieve real-time inference while maintaining high detection accuracy. These studies provide valuable insights and methodologies that inspire the development of charging port detection systems.

**Table 1:** Comparison of existing methods for EV charging Port detection and localization.

References	Key Features	Limitations	Performance
Zhang et al.^13^	HSI color model for segmentation; Median filtering for noise reduction; Morphological operations and Canny edge detection	Sensitive to extreme lighting; Limited robustness at long distances	Accuracy: 100% (under controlled conditions)
Tadic^8^	Depth sensor for 3D scene reconstruction; Morphological operations for object extraction; Simple image processing pipeline	Requires controlled illumination; Limited to CCS2 sockets	Detection rate: 94%
Zhao et al.^11^	Modified YOLOv4 for recognition; Meanshift clustering for noise removal; Affine correction for coordinate refinement	Requires large labeled dataset; Limited to indoor environment	Detection success rate: 100% Avg. processing time: 27ms
Mahhadevan et al.^12^	SWIN-Transformer for global context; SimAM attention mechanism	High computational complexity; Requires powerful hardware	mAP0.5: 81.4
FasterEVPoints	Integrating PConv, SE, and a modified PAN with FasterNet to balance speed and accuracy; PnP algorithm and BA optimization algorithm for Pose estimation; Modified NMS for post-processing	Potential inaccuracies for different charging ports; Limited adaptability under extreme conditions	Accuracy rate: 95%; mAP0.5:0.95: 33.3

Methods based on traditional image processing often struggle to maintain accuracy in complex and dynamic environments. However, with the advent of deep learning, convolutional neural networks (CNNs) have demonstrated remarkable performance in object detection tasks. This is exemplified by the two-stage region-based convolutional neural network (RCNN) series^22–24^ and the one-stage you only look once (YOLO) series^25–30^, both of which have been successfully applied to charging port recognition and localization. Zhao et al.^11^ introduced a modified YOLO version 4 (YOLOv4) model for charging port recognition, incorporating affine transformation for precise extraction. Although the model demonstrates promising results, there remains potential for improvement in terms of both speed and accuracy. Mahhadevan et al.^12^ proposed an advanced CNN model integrated with the SWIN-Transformer^31^ for charging port detection. While these deep learning-based methods significantly outperform traditional image processing techniques in terms of accuracy and stability, they still exhibit several limitations that warrant attention: (1) Current technique are limited to capturing the two-dimensional (2D) bounding box of the charging port in images, necessitating additional techniques to accurately determine its three-dimensional (3D) pose; (2) The proposed models are encumbered by an overabundance of parameters and substantial computational demands, presenting implementation challenges on economically viable edge devices.

**Fig. 1:**
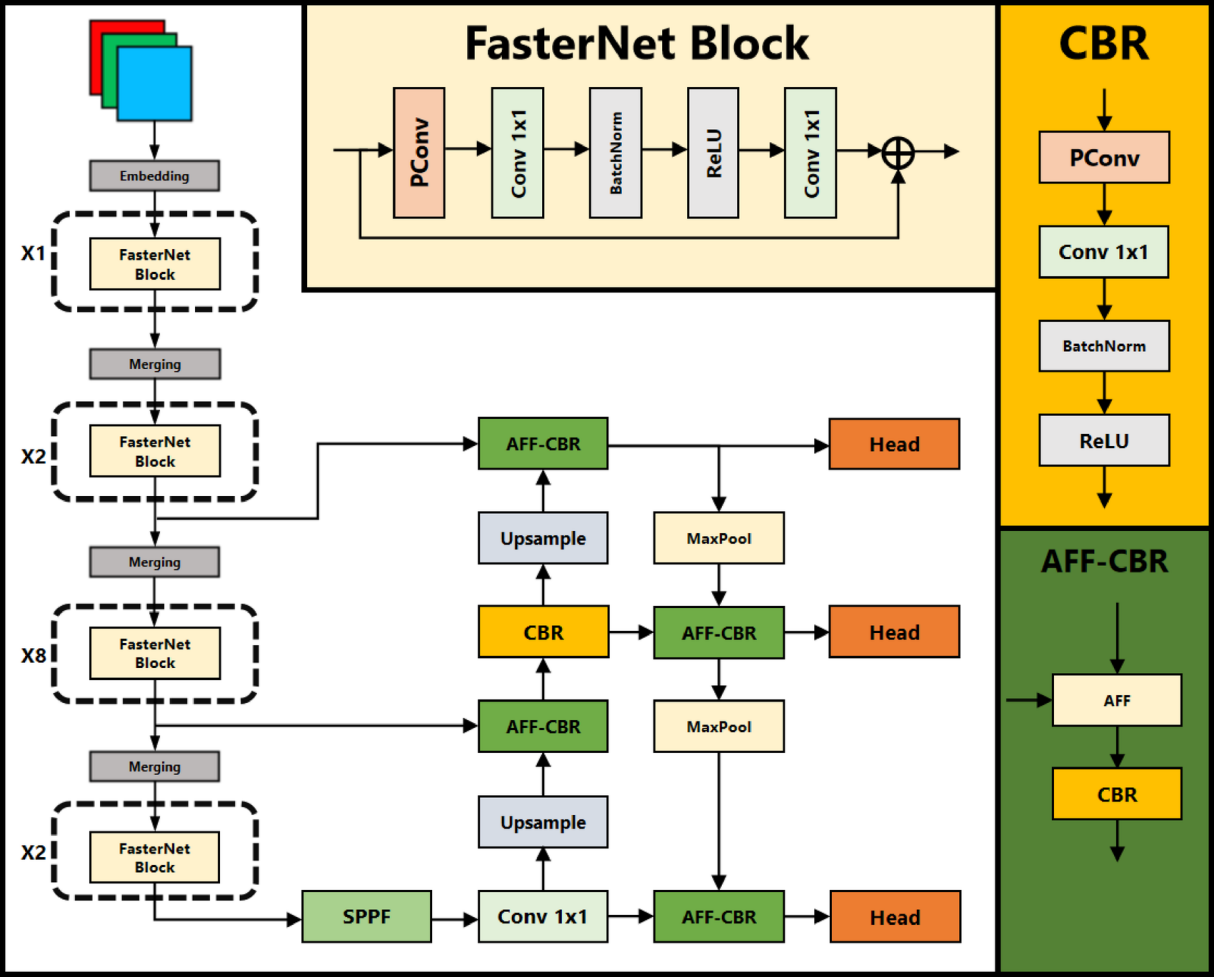
Architecture of the proposed FasterEVPoints model.

To address these challenges, we propose the FasterEVPoints CNN model, which directly predicts the coordinates of keypoints. Its overall architecture is illustrated in Fig. 1. This innovative approach enables the direct acquisition of the target’s pose through perspective-n-point (PnP) solving^32^, eliminating the need for additional sensors. FasterEVPoints is built upon the efficient partial convolution (PConv) and FasterNet modules^33^, offering exceptional computational efficiency and speed advantages. To further improve pose accuracy, we incorporated bundle adjustment (BA) optimization techniques for pose refinement. This approach highlights our commitment to leveraging the power of computer vision and deep learning methodologies to enhance the accuracy and efficiency of automated systems. The comparison of the proposed methodology with the existing methodology for EV charging port detection and localization is shown in Table 1. Our contributions are clearly outlined as follows:

1. **FasterEVPoints Model Development**: A novel CNN architecture that achieves an optimal balance between accuracy and inference speed by integrating PConv, FasterNet, squeeze-and-excitation (SE), and the path aggregation network (PAN) modules.

2 **Integration of the Attentional Feature Fusion (AFF) Module into PAN**: By incorporating the AFF module, the PAN^34^ module is enhanced to effectively integrate feature maps from multiple scales. This modification not only boosts the framework’s accuracy but also reduces the computational load, underscoring a commitment to efficient design.

3 **Innovative Keypoints Prediction for EV Charging Ports**: A CNN-based technique is employed to directly predict keypoints on EV charging ports, simplifying the identification process and eliminating the need for complex, error-prone traditional image processing methods. To further enhance the localization accuracy, the WingLoss function is utilized as the loss function during training.

4 **Enhancement of Pose Estimation Workflow**: Pose estimation techniques have been refined through the integrated use of PnP solving and multi-frame BA optimization. This approach provides a substantial boost in the accuracy and reliability of pose extraction, ensuring precise alignments even in highly complex setups.

These initiatives represent significant improvements over existing technologies, marking considerable advancements in both the functionality and practical applicability of automated systems.

### Proposed FasterEVPoints model

This section provides an overview of the PConv and FasterNet architecture, followed by a detailed introduction to the proposed FasterEVPoints model, specifically designed for keypoints detection in the context of EV charging port localization.

**Fig. 2:**
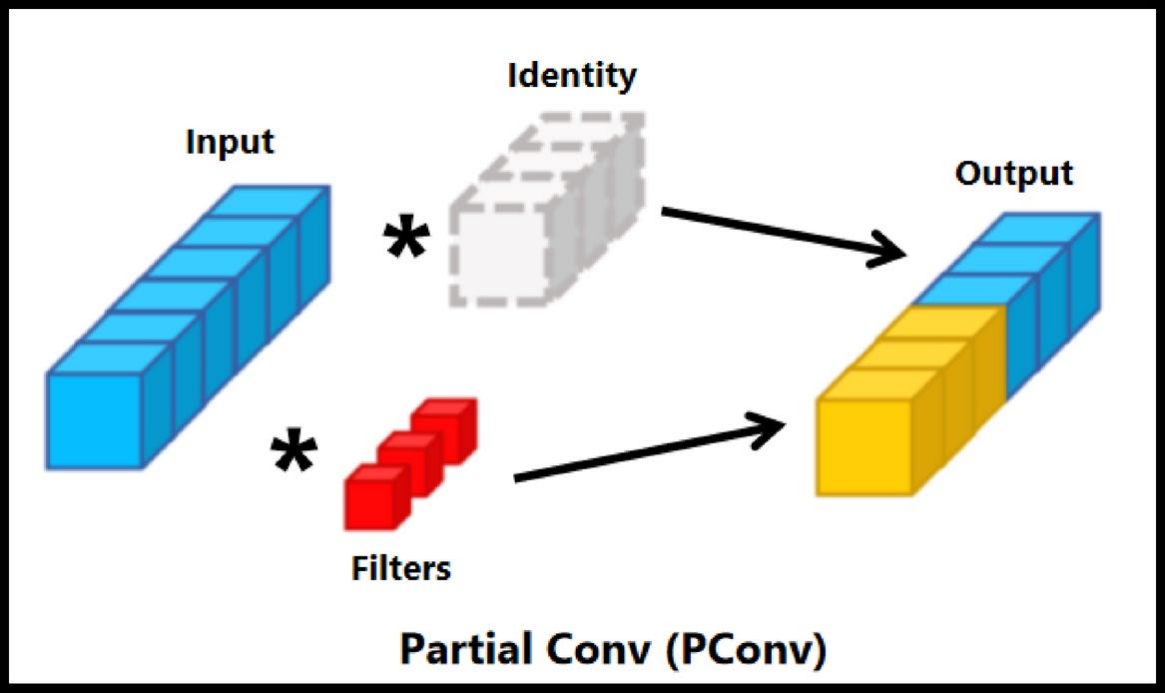
Partial convolution. It improves speed and efficiency by selectively applying filters to a specific subset of input channels.

### Basic model architecture

In many practical scenarios, CNN models must be deployed on platforms with limited computational and memory resources, such as edge or embedded systems. This constraint necessitates the adoption of lightweight CNN architectures. Typically, these architectures aim to minimize floating-point operations (FLOPs), with techniques like depthwise Conv (DWConv) being extensively employed due to their reduced FLOPs and parameter counts. Despite this reduction, the frequent memory accesses required by DWConv hinder its ability to fully leverage available computational resources, thereby decoupling the reduction in FLOPs from a proportional decrease in latency. The relationship between latency and FLOPs, as expressed in Eq. (1), highlights that while DWConv achieves a substantial reduction in FLOPs compared to standard convolutions, this does not necessarily lead to a corresponding improvement in processing speed.

1 $$Latency=\frac{{FLOPs}}{{FLOPS}}$$

To mitigate this issue, PConv applies convolution operation to on only a subset of channels, leaving the rest unchanged for the next layer^33^, as illustrated in Fig. 2. The feature information from these unchanged channels gradually propagates across all channels through a series of PConv layers. Integrating PConv with pointwise Conv (PWConv) efficiently leverages data across all channels. PConv achieves a higher FLOPS than DWConv while maintaining a lower FLOPs than standard Conv.

FasterNet-T0 is a tiny CNN backbone built upon PConv, delivering remarkable performance in both speed and accuracy. Its architecture is shown in Fig. 1. FasterNet-T0 consists of four stages, each comprising multiple interconnected FasterNet Blocks. Each FasterNet Block begins with a PConv layer followed by two PWConv layers. The output from this sequence undergoes a residual connection^35^ with the initial input before producing the final output. Between successive FasterNet Blocks, a stride-2 convolution serves as a merging layer, reducing the feature map resolution to lower the parameter counts and computational cost.

### FasterEVPoints model architecture

This section presents FasterEVPoints, a pioneering single-stage, anchor-free, lightweight CNN model designed for rapid and precise keypoints prediction of EV charging ports. The architecture of FasterEVPoints comprises three primary components: the Backbone, Neck, and Head, each playing a crucial role in its high-performance capabilities, as depicted in Fig. 1.

The Backbone of FasterEVPoints is built upon the FasterNet-T0 architecture, chosen for its optimal balance between speed and accuracy. To further enhance feature recalibration and focus, SE modules^36^ are incorporated, marking a significant improvement over the conventional FasterNet design. This architectural refinement reflects our commitment to advancing detection capabilities. Complementing the Backbone, the spatial pyramid pooling fusion (SPPF) module^28^ is adopted, exemplifying a tailored approach to sophisticated feature processing. The Neck employs a modified PAN, an enhanced design that enables more effective multi-scale feature integration compared to its original counterpart. This modification highlights the custom innovations embedded within the FasterEVPoints model. By leveraging PConv, the parameter count and computational complexity are notably reduced, allowing for the adoption of larger convolutional kernel sizes in the Neck and Head. This optimization enhances overall network performance without imposing excessive computational overhead. The Head section features a decoupled design strategy optimized specifically for direct keypoints prediction, diverging from traditional bounding box-based detection methods. This approach enables precise identification of EV charging port keypoints without additional image processing, thereby streamlining the entire detection pipeline. For post-processing, a specialized variant of the non-maximum suppression (NMS) algorithm is implemented, adapted to handle the bounding rectangle of predicted points rather than standard bounding box. By applying weighted summation, this method effectively minimizes jitter in keypoints, leading to greater accuracy and reliability. The following subsections (1) to (5) provide a comprehensive and systematic analysis of each component.

**Fig. 3:**
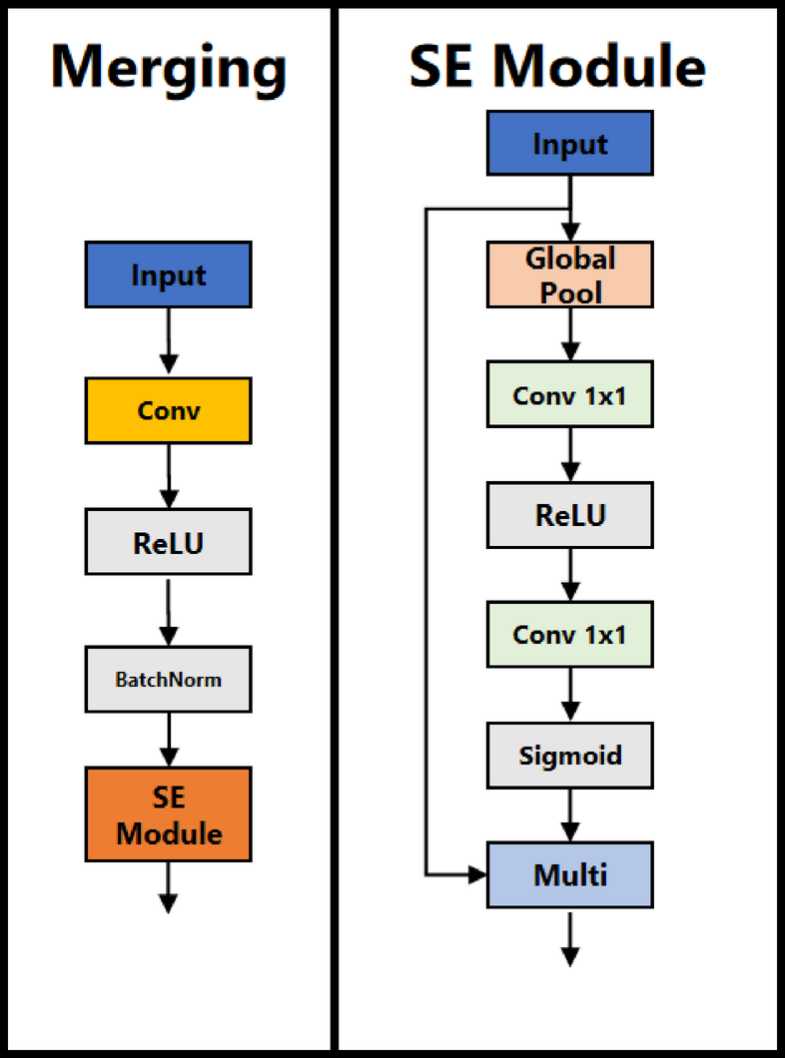
Structure of “squeeze-and-excitation” (SE) module, along with the updated merging layer, is employed to downsample the feature map prior to its input to the subsequent stage of the FasterNet backbone.

1. **FasterNet Integrated with SE Modules**: A pivotal enhancement in contemporary CNN architectures compared to earlier designs is the improved facilitation of information exchange across channels. Despite FasterNet achieves cross-channel interaction primarily through pointwise convolutions, its simplicity comes at the cost of lacking mechanisms such as Channel Shuffle in ShuffleNet^37^, which facilitates channel mixing without increasing parameter complexity. This limitation could hinder effective information propagation across channels. Mitigating this constraint by increasing the number of FasterNet Blocks or employing denser pointwise convolutions would, however, result in an unsustainable growth in parameters.
  To overcome this challenge, the SE module is incorporated into FasterNet, enabling adaptive feature re-weighting through squeeze-and-excitation operations, a mechanism known as channel attention. The squeeze operation utilizes a global average pooling (GAP) layer to condense spatial information within each channel into a single scalar descriptor, capturing the global context of the feature map. Mathematically, for the input feature map $X \in {{\mathbb{R}}^{H \times W \times C}}$, the squeezed feature vector $Z \in {{\mathbb{R}}^C}$ is formulated in Eq. (2), where ${X_c}\left( {i,j} \right)$represents the activation at spatial location $\left( {i,j} \right)$ in a specific channel. Subsequently, the excitation operation applies two fully connected (FC) layers, replaced with two $1 \times 1$ convolutions, to model intricate channel-wise dependencies. The transformation expressed in Eq. (3), where ${W_1}$ and ${W_2}$​ are learnable weight matrices, $\delta$ denotes the rectified linear unit (ReLU) activation, and $\sigma$ is the sigmoid function. Finally, recalibrated feature vector* s* is used to reweight the original feature map through channel-wise multiplication, as formulated in Eq. (4).
  2 $${z_c}=\frac{1}{{H \times W}}\sum\nolimits_{{i=1}}^{H} {\sum\nolimits_{{j=1}}^{W} {{x_c}(i,j)} }$$
  3 $$s=\sigma \left( {{W_2}\delta \left( {{W_1}z} \right)} \right)$$
  4 $$\widetilde {{{X_c}}}={s_c}{X_c}$$
  By integrating the SE module into FasterNet, the network dynamically adjusts channel-wise feature importance, enhancing feature extraction efficiency without introducing excessive parameter growth. The SE module is applied to the merging layers of all stages except the first, reinforcing the network’s capacity to capture essential features across different levels of abstraction. The updated merging layer structure is depicted in Fig. 3.

2 **Modified PAN**: In the FasterEVPoints model, a modified PAN serves as the architectural neck. This enhanced feature pyramid network features a dual-directional information flow, utilizing both top-down and bottom-up pathways to ensure thorough integration of features across various scales. A significant enhancement in this modified PAN is the strategic replacement of the conventional CSPLayer with the AFF module^38^, bolstering the model’s capability for more effective feature integration, as illustrated in Fig. 1.
  The AFF module, an innovation by Yimian Dai et al.^38^, introduces a channel attention-driven mechanism for feature fusion. Given two input feature maps $A,B \in {{\mathbb{R}}^{H \times W \times C}}$ from different scales or network stages, the module first applies a summation operation, as expressed in Eq. (5). Meanwhile, to capture global contextual dependencies,* F* is processed through GAP, generating a global descriptor $g\left( F \right)$, as formulated in Eq. (6). This operation encodes spatially invariant global information, allowing the model to dynamically determine channel-wise importance scores. The concatenated features and global descriptor undergo two sets of $1 \times 1$ convolutions, each followed by batch normalization (BatchNorm) and ReLU activation, refining the feature representations as described in Eqs. (7) and (8).
  5 $$F=A+B$$
  6 $$g\left( F \right)=\frac{1}{{H \times W}}\sum\nolimits_{{i=1}}^{H} {\sum\nolimits_{{j=1}}^{W} {F(i,j)} }$$
  7 $$\hat {F}=\operatorname{Re} LU\left( {BN\left( {Con{v_{1 \times 1}}\left( F \right)} \right)} \right)$$
  8 $$\hat {g}\left( F \right)=\operatorname{Re} LU\left( {BN\left( {Con{v_{1 \times 1}}\left( {g\left( F \right)} \right)} \right)} \right)$$

The transformed features are then aggregated and passed through a sigmoid function to compute the channel-wise attention weight $\alpha$.The final fused feature map is computed as follows:

9 $$Y=\alpha \cdot A+\left( {1 - \alpha } \right) \cdot B$$

**Fig. 4:**
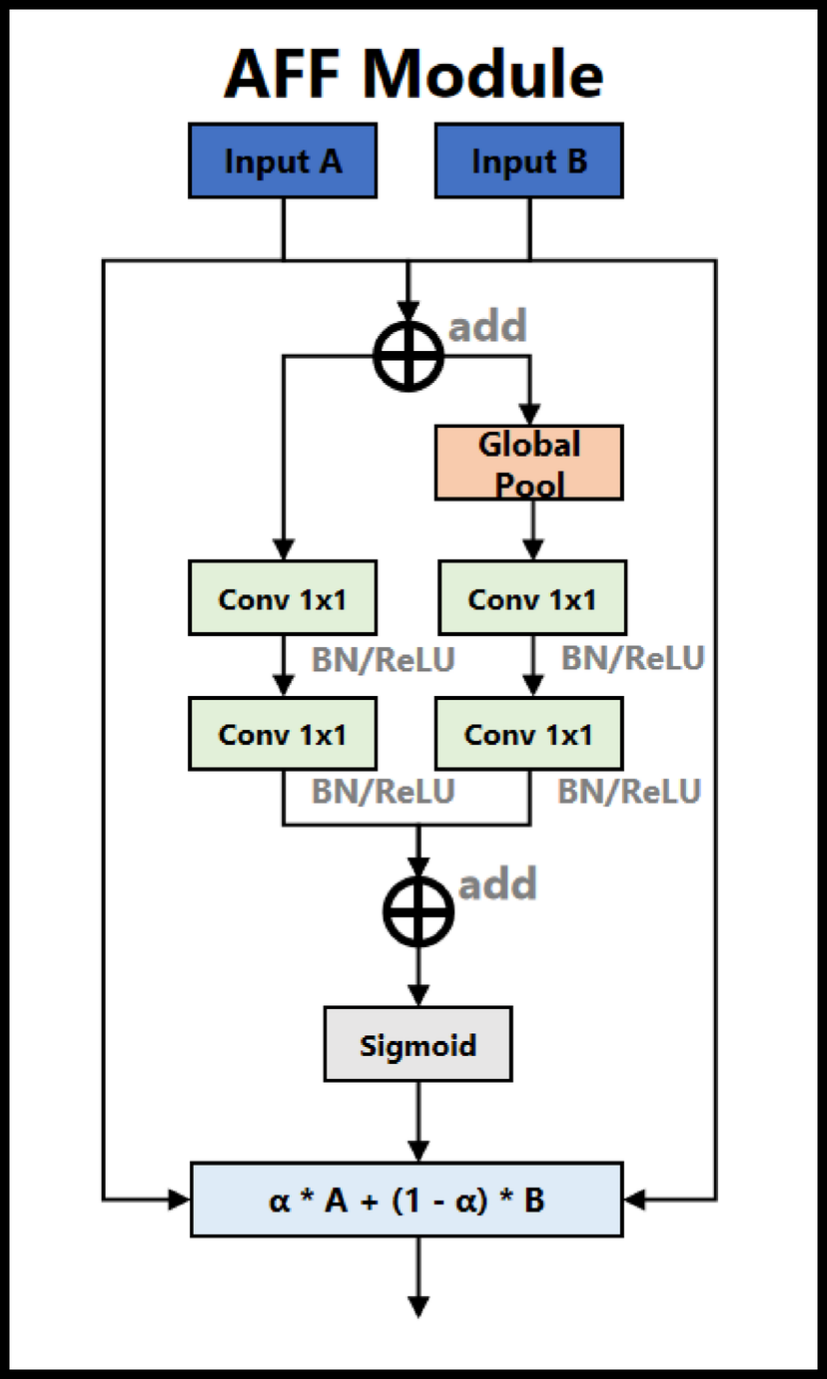
Structure of attention feature fusion module (AFF).

Designed to supplant traditional methods of concatenation and addition, which typically provide only fixed, linear aggregation of features, the AFF module offers a dynamic and adaptive approach. It intelligently weights and amalgamates features from different layers, addressing the challenges of scale discrepancy and semantic inconsistency among feature maps from various depths of the network. This method not only preserves but also enriches the semantic integrity of the fused features, ensuring a more coherent integration. The architecture of the AFF module is detailed in Fig. 4.

3 **Larger Kernel Size**: In conventional convolutional operations, the computational cost, expressed in FLOPs, is determined as shown in Eq. (10). Here,* w* and* h* represent the width and height of the input,* c* is the number of channels, and* k* is the kernel size. By contrast, PConv reduce the FLOPs to the form presented in Eq. (11), where ${c_p}\left( { \leqslant c} \right)$ is the number of channels for spatial feature extraction. Notably, when the ratio ${{{c_p}} \mathord{\left/ {\vphantom {{{c_p}} c}} \right. \kern-0pt} c}$ equals 1/4, the FLOPs of PConv with the same kernel size amounts to only 1/16 of that for regular convolution.
  10 $$FLOPs\left( {Conv} \right)=w \cdot h \cdot {c^2} \cdot {k^2}$$
  11 $$FLOPs\left( {PConv} \right)=w \cdot h \cdot {c_p} \cdot {k^2}$$
  This substantial reduction in computational demand facilitates the use of larger convolutional kernel sizes to expand the receptive field, thereby enhancing the network’s capability for feature extraction. In the FasterEVPoints model, PConvs with $5 \times 5$ kernels are utilized at the neck and head to leverage these benefits.

**Fig. 5:**
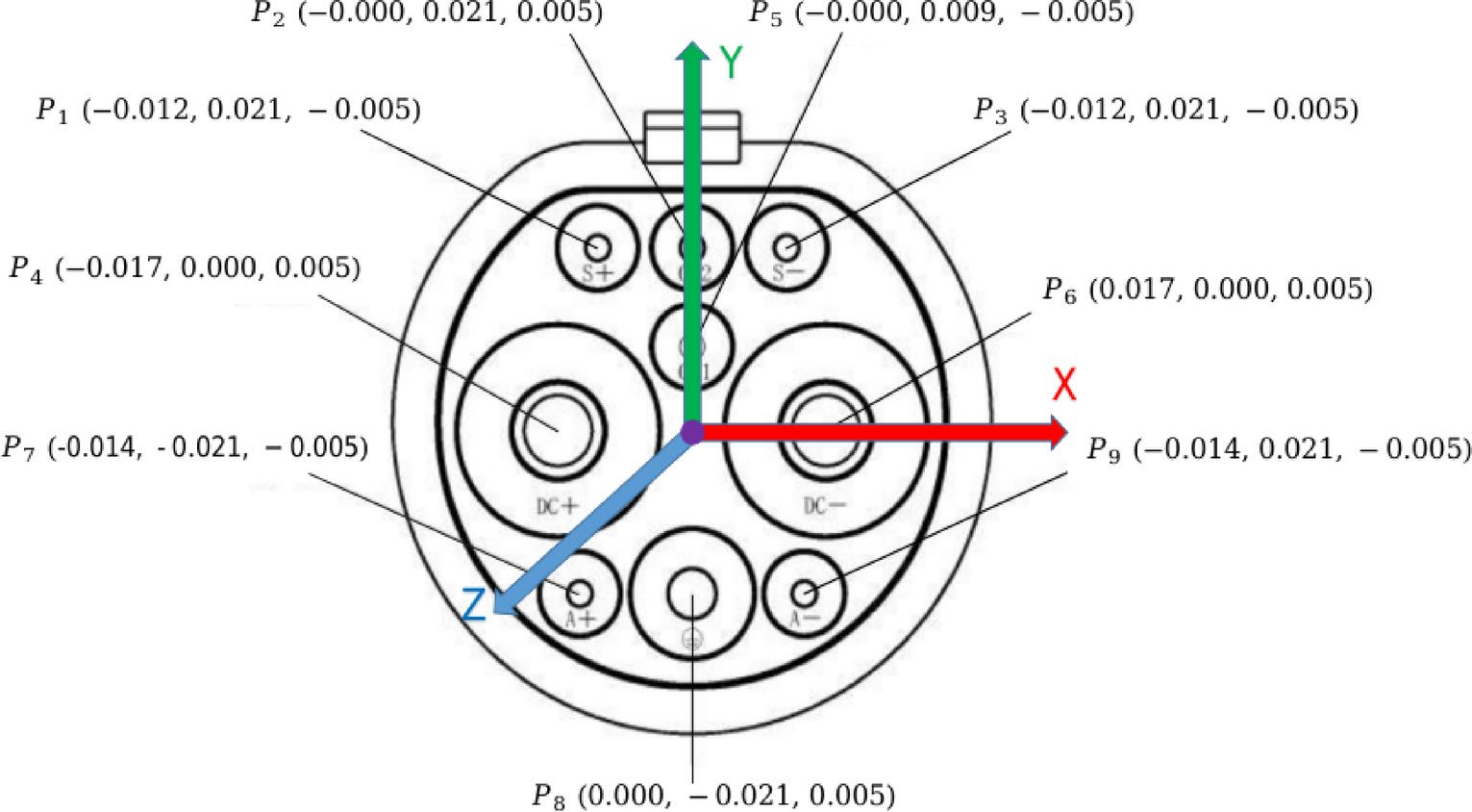
Display of 9 keypoints and their corresponding 3D coordinates for EV charging ports.

**Fig. 6:**
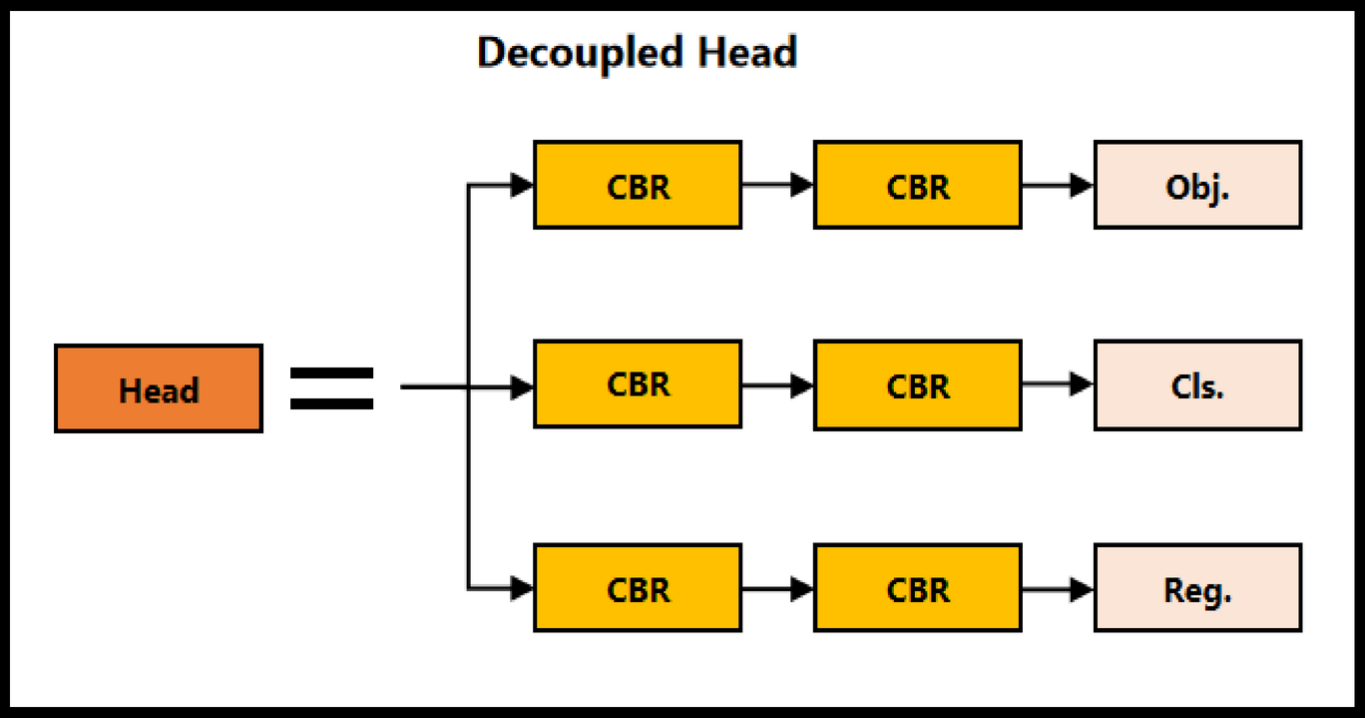
Decoupled head of FasterEVPoints consists of three distinct branches, each branch encompassing two convolutional layers that are seamlessly integrated with BatchNorm and ReLU activation functions.

4 **Keypoint Regression**: The goal of FasterEVPoints model is to accurately extract keypoints from EV charging ports for solving PnP problems. Employing CNNs for direct keypoints prediction enhances robustness under challenging and dynamic lighting conditions, surpassing traditional image processing-based machine vision techniques. As illustrated in Fig. 5, the keypoints of an EV charging port are identified as the centers of the 9 contact areas. WingLoss is utilized as the loss function for keypoints regression, offering advantages over Smooth L1 and L2 loss by providing a larger gradient for small errors, thus facilitating more precise keypoints prediction. The formulation for WingLoss is detailed in Eqs. (12) and (13), where $w=10$, $e=2$, and* C* is derived as per Eq. (14). The variables ${s_i}$ and ${s_i}\prime$ represent the predicted and ground truth keypoints, respectively.
  12 $$wing\left( x \right)=\left\{ {\begin{array}{*{20}{l}} {w \cdot \ln \left( {1+\frac{{\left| x \right|}}{e}} \right) \leqslant 0}&{{\text{if }}x<w,} \\ {\left| x \right| - C}&{{\text{otherxwise}}{\text{.}}} \end{array}} \right.$$
  13 $$WingLoss=\sum\limits_{i} {wing\left( {{s_i} - {s_i}\prime } \right)}$$
  14 $$C=w - w \cdot \ln \left( {1+\frac{w}{e}} \right)$$
  The head of the FasterEVPoints model features a simple decoupled design, as depicted in Fig. 6. Each head consists of three parallel branches, each equipped with two $5 \times 5$ convolutional layers for object classification, class classification, and keypoints regression, all integrated with BatchNorm and ReLU.

**Figure:**
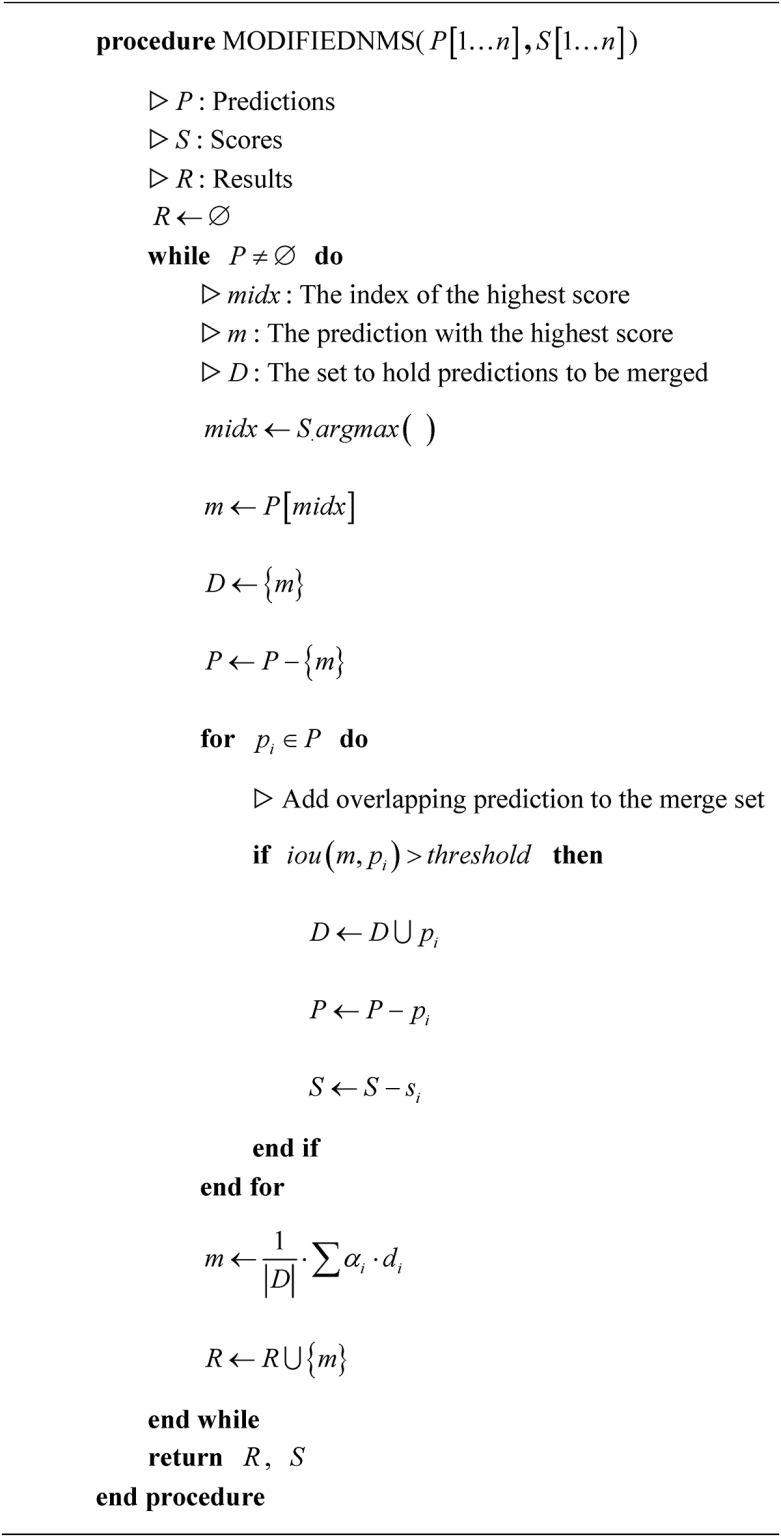
**Algorithm 1** Detailed illustration of the modified NMS algorithm

5 **Post-processing and Modified NMS**: The FasterEVPoints model uniquely predicts keypoints of EV charging ports directly, eliminating the need for conventional bounding box data. To adapt the NMS algorithm to this approach, the bounding box traditionally used in NMS is replaced with the minimum bounding rectangle that encloses the predicted keypoints, facilitating the calculation of intersection over union (IoU).
  Traditional NMS algorithms typically prioritize the prediction with the highest classification confidence to determine the target’s location, often disregarding lower-confidence predictions that may contain valuable information. This approach can result in inconsistent keypoint localization, due to the exclusion of potentially relevant data. To address this limitation, an enhanced NMS algorithm is introduced, as detailed in Algorithm 1. This modification incorporates information from all predictions, improving the stability and precision of keypoint localization. In Algorithm 1, each iteration begins by identifying the prediction with the highest score. The algorithm then evaluates the overlap of remaining predictions using the IoU metric. Predictions exhibiting substantial overlap are fused into a single output via weighted averaging, with weights typically assigned based on the original confidence scores. By comprehensively considering all significant predictions, the algorithm mitigates reliance on a single high-confidence prediction, yielding a more balanced and robust estimate. This approach markedly improves the accuracy and consistency of keypoint localization, ensuring more reliable results.

### Pose extraction and correction algorithm

This section introduces extended perspective-n-point (EPnP) and BA algorithms, and then proposes an inter-frame matching strategy to formulate a comprehensive multi-frame optimization framework.

### EPnP algorithm for solving initial pose

**Fig. 7:**
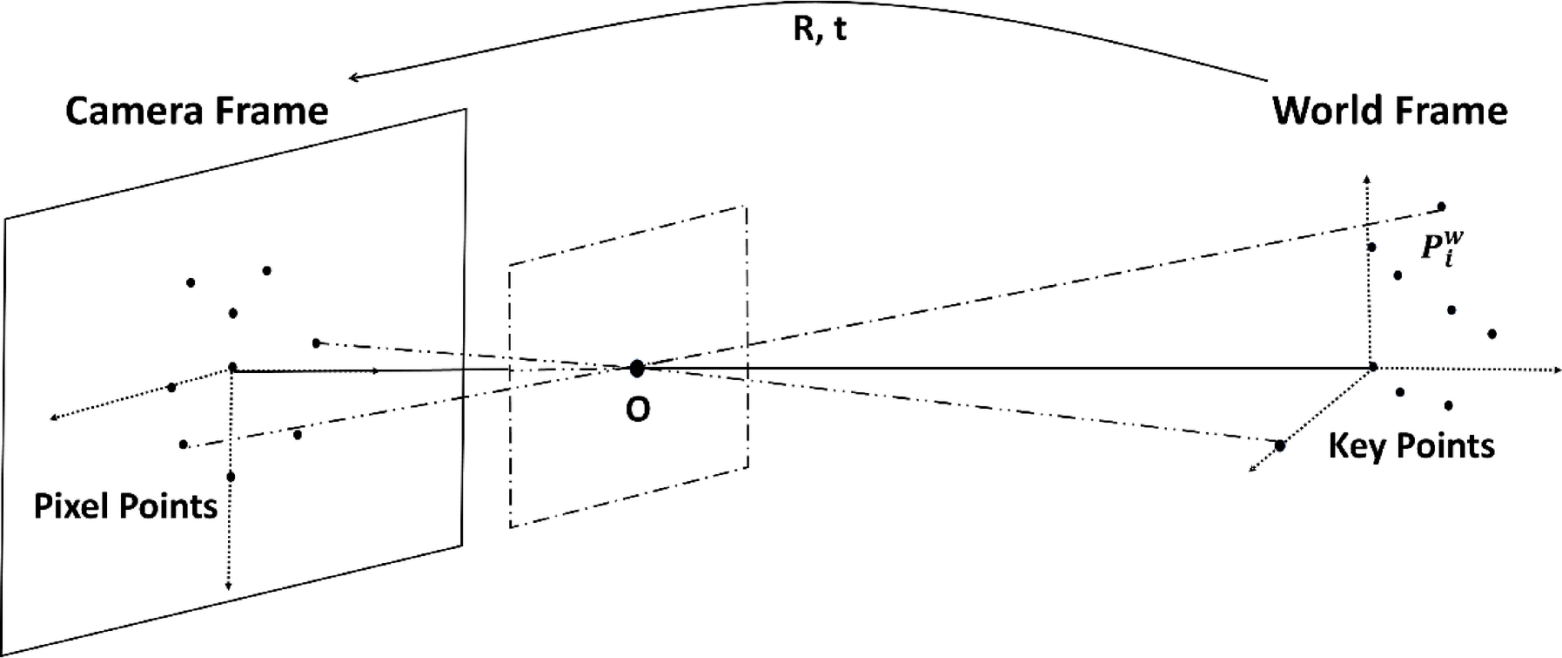
The schematic of the PnP algorithm that solves for the camera coordinate system’s position relative to the world coordinate system.

In the context of computer vision and 3D reconstruction, the PnP algorithms play a vital role in tasks such as camera localization and pose estimation. The PnP algorithm is specifically designed to tackle the challenge of accurately determining a camera’s pose (both its position and orientation) relative to the world coordinate system. Its schematic is shown in the Fig. 7. This determination is achieved by leveraging the correspondence between 3D points in the real-world environment and their corresponding 2D projections captured within the camera image.

The EPnP algorithm distinguishes itself as a non-iterative and highly efficient solution that exhibits robustness against noise and uncertainty in the input data^32^. Its non-iterative nature not only ensures computational efficiency but also renders it well-suited for real-time applications. In the context of the EV charging port, we have successfully obtained the key pixel points that correspond to their actual spatial positions. Leveraging these known 3D-2D point correspondences, EPnP can efficiently estimate the initial position and orientation of the charging port within the camera coordinate system.

A fundamental prerequisite for the implementing the algorithm is the establishment of a corresponding spatial coordinate representation. The world frame is defined as ${\left( \cdot \right)^w}$, with the geometric center of the charging port serving as the origin of the world coordinate system. Within this system, the world coordinates of the keypoints, as depicted in Fig. 5, are denoted as $P_{i}^{w}$, where* i* signifies the index of each individual keypoint. Mathematically, EPnP optimizes pose estimation by representing each 3D point $P_{i}^{w}$​ as a linear combination of four predefined control points $c_{j}^{w}$:

15 $$P_{i}^{w}=\sum\limits_{{j=1}}^{4} {{\alpha _{i{\text{ }}j}}} c_{j}^{w},{\text{ with}}\sum\limits_{{j=1}}^{4} {{\alpha _{i{\text{ }}j}}} =1$$

This transformation reduces the number of unknowns, requiring only the estimation of the camera coordinates of the four control points instead of all 3D points. Given the camera pose $\left( {R,{\text{ }}t} \right)$, the 3D points in the camera frame are expressed as:

16 $$P_{i}^{c}=RP_{i}^{w}+t=\sum\limits_{{j=1}}^{4} {{\alpha _{i{\text{ }}j}}} c_{j}^{c}$$

Using the intrinsic camera matrix* K*, the perspective projection of these 3D points onto the image plane follows Eq. (17). Expanding* K*, two equations for the pixel coordinates $\left( {{u_i},{\text{ }}{v_i}} \right)$are derived, as shown in Eq. (18). These linear equations can then be rewritten in matrix form, as expressed in Eq. (19).

17 $$\forall i,{\text{ }}{w_i}\left[ {\begin{array}{*{20}{c}} {{u_i}} \\ {{v_i}} \\ 1 \end{array}} \right]=KP_{i}^{c}=K\sum\limits_{{j=1}}^{4} {{\alpha _{i{\text{ }}j}}} \left[ {\begin{array}{*{20}{c}} {x_{j}^{c}} \\ {y_{j}^{c}} \\ {z_{j}^{c}} \end{array}} \right],{\text{ with }}K=\left[ {\begin{array}{*{20}{c}} {{f_x}}&0&{{c_x}} \\ 0&{{f_y}}&{{c_y}} \\ 0&0&1 \end{array}} \right]$$

18 $$\begin{array}{*{20}{c}} {\sum\limits_{{j=1}}^{4} {{\alpha _{i{\text{ }}j}}} {f_u}x_{j}^{c}+{\alpha _{i{\text{ }}j}}\left( {{u_c} - {u_i}} \right)z_{j}^{c}=0} \\ {\sum\limits_{{j=1}}^{4} {{\alpha _{i{\text{ }}j}}} {f_v}y_{j}^{c}+{\alpha _{i{\text{ }}j}}\left( {{v_c} - {v_i}} \right)z_{j}^{c}=0} \end{array}$$

19 $$\mathbf{M}\mathbf{x}=0$$

Among them, ${w_i}$ are scalar projective parameters, $\mathbf{x}$ is a 12-dimensional vector of unknowns, and $\mathbf{M}$ is a $2n \times 12$ matrix constructed by organizing the coefficients of Eq. (18) for each reference point. The camera position is efficiently estimated by solving the singular value decomposition (SVD) decomposition. In this study, given the known camera intrinsic parameters and corresponding image pixel points, the initial pose of the charging port is determined using the EPnP algorithm, seamlessly integrated within the open source computer vision (OpenCV) library^39^.

### BA optimization and inter-frame matching

1. **BA Optimization Algorithm**: The precise pose acquisition of the charging port is crucial for autonomous EV charging. Although the EPnP algorithm is utilized as a valuable initial estimator, it may not always meet the stringent accuracy requirements. To enhance the initial pose estimation, the BA algorithm is employed to minimize reprojection error by formulating a nonlinear least squares problem. This is achieved by iteratively optimizing the discrepancies between observed and reprojected poses. Through this process, the BA algorithm enhances the precision of the initial pose estimation, greatly improving the efficiency of EV charging. The symbol annotation is the same as the Sect. 3.1, where $P_{i}^{w}$ represents the* i*-th keypoint in world frame. As depicted in Fig. 5, the keypoints in the world coordinate system are accurately determined. Subsequently, each keypoint is reprojected using the refined pose estimation, denoted as ${{\text{x}}_i}$. The expression of ${x_i}$ is outlined in Eq. (20).
  20 $${x_i}=\left[ {\begin{array}{*{20}{c}} {{u_i}} \\ {{v_i}} \end{array}} \right]$$
  FasterEVPoints algorithm accurately predicts the key pixel points of the EV charging port, denoted as ${z_i}$. Since the rotation matrix is not closed under addition and cannot be directly derived, pose variables requiring are expressed using Lie algebra, denoted as $\upxi$. This representation enables the formulation of a least-squares optimization problem, whose mathematical expression is as follows:
  21 $$\hbox{min} {\text{ }}f\left( {\upxi ,P_{1}^{w} \ldots P_{9}^{w}} \right)=\hbox{min} \sum\limits_{{i=1}}^{9} {\left( {{z_i} - {x_i}} \right)}$$

2 **Multi-frame Optimization Based the Matching Tracking Strategy**: In the actual working environment, the charging port typically appears only once in each frame captured by the camera. Given that continuous tracking of the same charging port is crucial during the actual charging process, we adopt a matched tracking strategy for multi-frame BA optimization to achieve better optimization results. Specifically, assuming that one EV charging port, denoted as ${s_{k - 1}}$, is recognized at the time ${t_{k - 1}}$. Subsequently, at the next time ${t_k}$, another charging port ${s_k}$ is recognized. The corresponding initial poses are expressed as ${\upxi _{k - 1}}$ and ${\upxi _k}$ using Lie algebra, respectively. If the constraints defined in Eq. (22), Eq. (23) and Eq. (24) are satisfied between these two pockets, ${s_{k - 1}}$ and ${s_k}$ are considered to be the same charging port, indicating a successful match.
  22 $$distance\left( {{s_k},{s_{k - 1}}} \right)<thres{h_d}$$
  23 $${t_k} - {t_{k - 1}}<thres{h_t}$$
  24 $$class\left( {{s_k}} \right)=class\left( {{s_{k - 1}}} \right)$$
  $thres{h_d}$ and $thres{h_t}$ represent the maximum distance threshold and maximum interval time threshold, respectively. Equation (24) serves as an indicator for identifying the same type of charging port.
  Based on this tracking strategy, we obtain a sequence of ten consecutive frames depicting the pose of the charging port. According to Eq. (21), the reprojection error constructed for each frame of charging port is denoted as ${e_k}$. Subsequently, the BA optimization problem for this sequence can be formulated as outlined in Eq. (25).
  25 $${\hat {\upxi }_1} \ldots {\hat {\upxi }_{10}},\hat {P}_{1}^{w} \ldots \hat {P}_{9}^{w}=argmin\sum\limits_{{k=1}}^{{10}} {f\left( {{\upxi _k},P_{1}^{w} \ldots P_{9}^{w}} \right)}$$
  ${\hat {\upxi }_i}$ represents the optimized camera pose in the* i*-th frame, expressed using Lie algebra, while $\hat {P}_{i}^{w}$ denotes the optimized 3D position of the charging port in the world coordinate system.
  We then employ the open-source nonlinear optimization library g2o^40^ to express the BA optimization problem as a graph structure, facilitating its resolution. Specifically, the Levenberg-Marquardt algorithm^41^ is utilized to solve the optimization problem. The optimized pose from the final frame serves as the current charging port pose.

## Experiments and results

### Experimental setting and evaluation metrics

1. **Experimental Settings**: The experiments were conducted on a computing system equipped with an NVIDIA GeForce RTX 3060 Laptop graphics processing unit with 6GB of graphic memory. The system operated on Ubuntu 22.04, with Python 3.10 and C + + 17 serving as the software development framework. The network training and evaluation were performed using the PyTorch deep-learning library (version 1.13) and CUDA (version 11.8). The EPnP algorithm was implemented via the solvePnP function in OpenCV (version 4.5.2), and the BA algorithm was executed using the g2o library.
  Python was employed for model training, whereas the algorithm deployment phase transitioned to C + + and its associated libraries. This strategic shift leveraged the computational efficiency of C + + for real-time execution. A comprehensive package for charging interface detection and localization was developed, seamlessly integrated into the ROS2 Humble framework.

2 **Evaluation Metrics**: In pose estimation tasks, evaluation metrics primarily encompass two aspects: position estimation error and attitude estimation error. Absolute distance serves as the measurement criterion for both. Position estimation error is quantified along the X, Y, and Z axes, while attitude estimation error is assessed in terms of Roll, Pitch, and Yaw orientations.
  To assess the detection capabilities of the enhanced model, a comprehensive set of evaluation metrics is employed, including precision, recall, mean average precision (mAP) at IoU thresholds of 0.5 and 0.5:0.95, total parameter count, model footprint, and processing speed. Certain metrics are defined based on the following concepts: true positives (TP, correctly classified as positive), false positives (FP, incorrectly classified as positive), and false negatives (FN, incorrectly classified as negative).
  Precision, as defined in Eq. (26), quantifies the proportion of correctly predicted positive instances relative to the total instances classified as positive by the model.
  26 $$Precision=\frac{{TP}}{{TP+FP}}$$
  Recall measures the ratio of actual positive samples correctly identified by the model, computed as delineated in Eq. (27).
  27 $$Recall=\frac{{TP}}{{TP+FN}}$$
  Average precision (AP) characterizes the area under the precision-recall curve, with its computation method specified in Eq. (28). The discrete formulation is provided in Eq. (29), where* K* represents the number of classes and* N* denotes the number of detected objects.
  28 $$AP=\int_{0}^{1} {Precision} \left( {Recall} \right)dRecall$$
  29 $$AP=\frac{1}{N}\sum\limits_{{k=1}}^{n} {Precision\left( k \right)} \times \Delta Recall\left( k \right)$$
  Meanwhile, mAP is defined as the weighted average of AP values across different categories, offering a holistic evaluation of the model’s detection performance across all classes, as detailed in Eq. (30). In this equation, $A{P_i}$ signifies the AP value for each category* i*, while represents the total number of categories in the training dataset. The metrics mAP0.5 and mAP0.5:0.95 indicate the model’s average detection accuracy at an IoU threshold of 0.5 and across an IoU range of 0.5 to 0.95, sampled at 0.05 intervals, respectively.
  30 $$mAP=\frac{1}{N}\sum\limits_{{i=1}}^{N} {A{P_i}}$$

### Dataset preparation

**Fig. 8:**
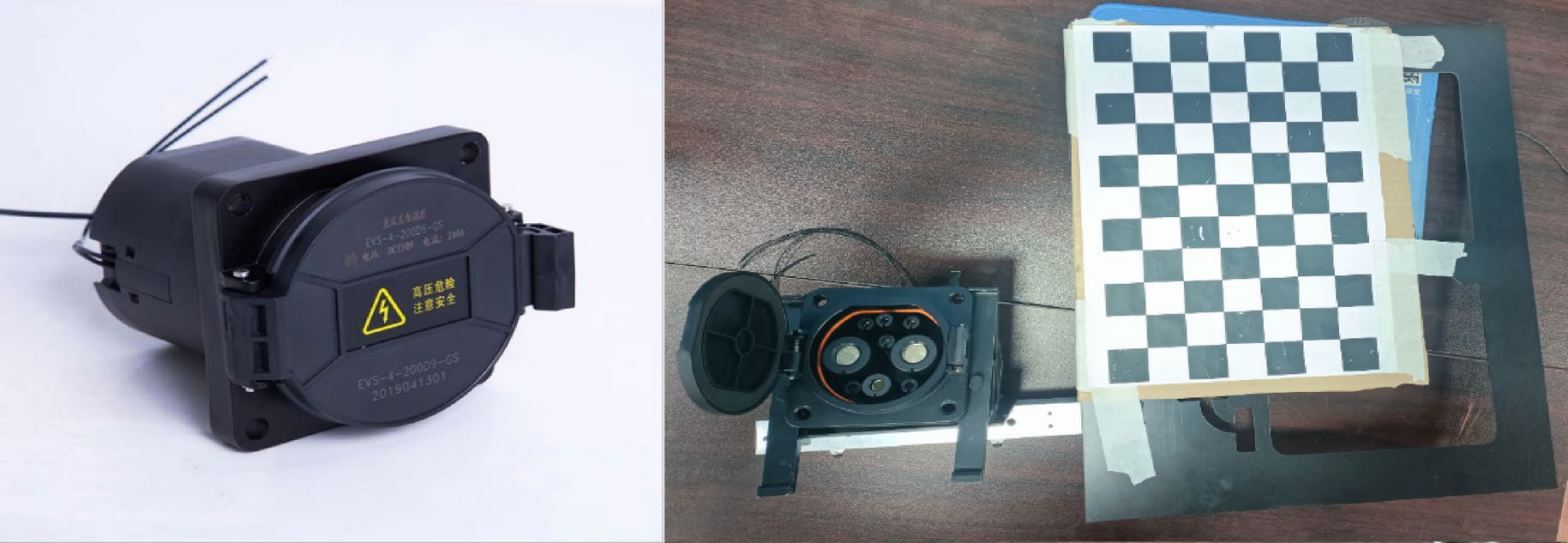
GB/T 20,234 DC charging port and self-made calibration device.

**Fig. 9:**
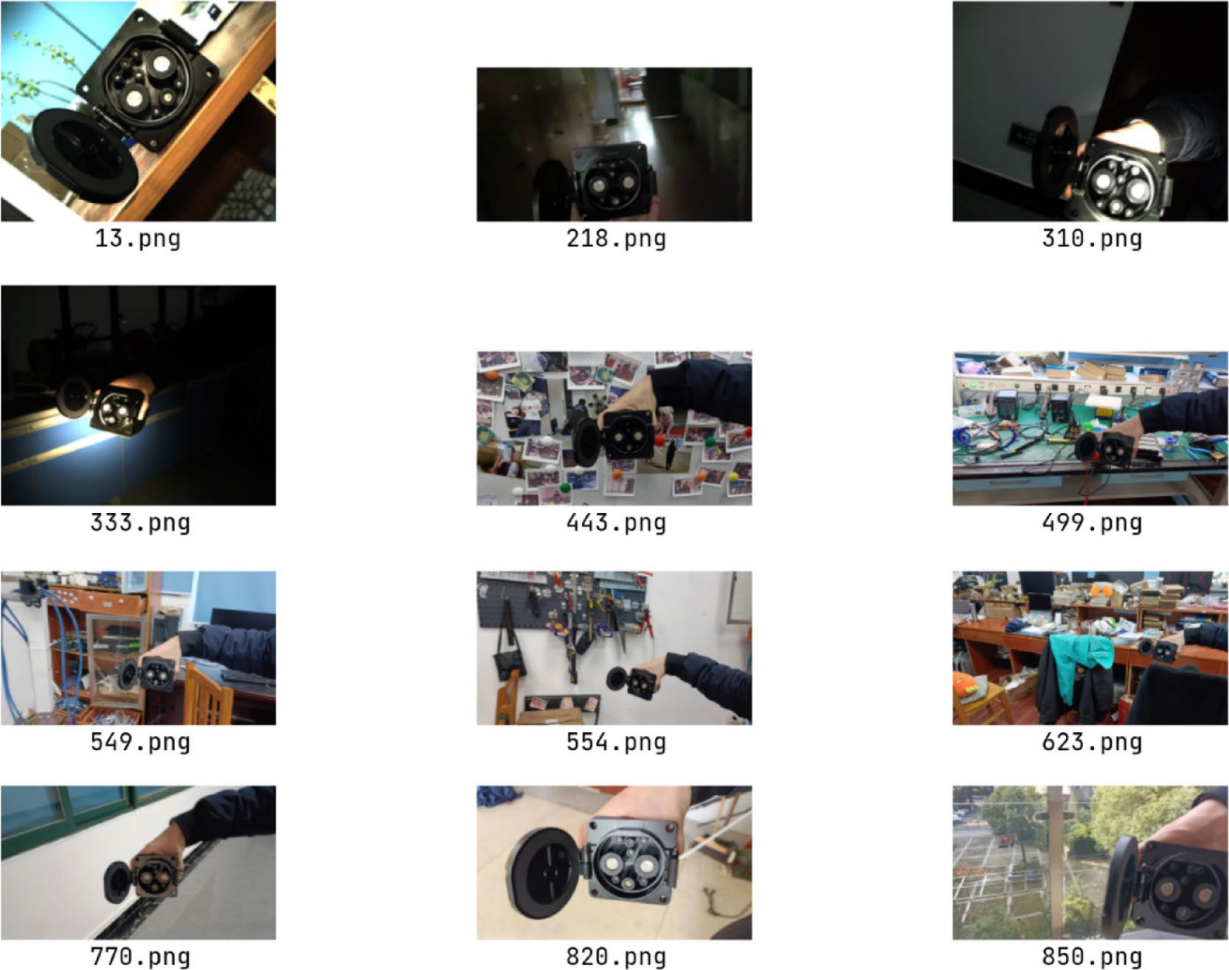
Partial images in the self-made charging port dataset.

1. **Datasets Collection**: Photographs of the National standard GB/T 20234 DC charging port were captured utilizing a Daheng Galaxy Mer2 industrial camera and a Xiaomi 10s smartphone, as shown in Fig. 8. These images were acquired from various distances, in diverse environments, and under varying lighting conditions, including natural sunlight, indoor illumination, and direct flashlight glare. The training dataset comprises approximately 1600 images, while the validation dataset consists of around 400 images. A selection of these images is presented in Fig. 9, providing a visual representation of the dataset.

**Fig. 10:**
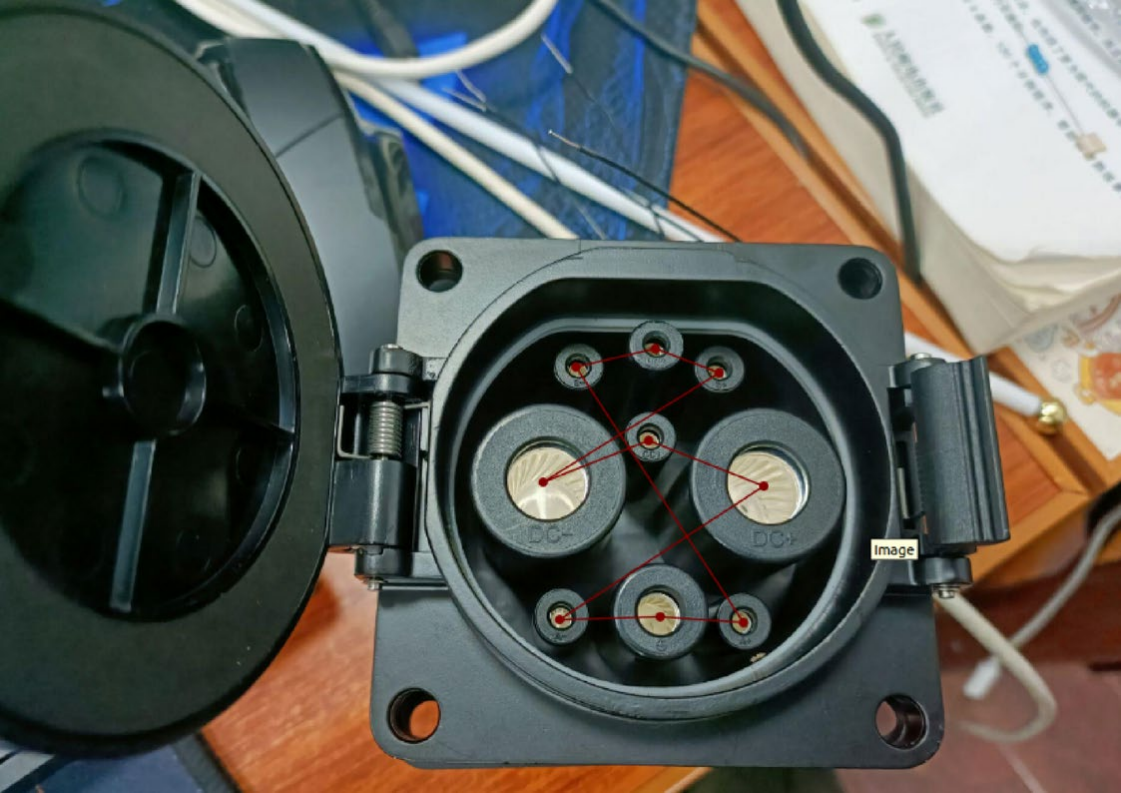
Screenshot of a labeled charging port obtained using Labelme. The intersecting lines visible in the image extend from ${P_1}$, located in the top left corner, to ${P_9}$, situated in the bottom right corner.

**Fig. 11:**
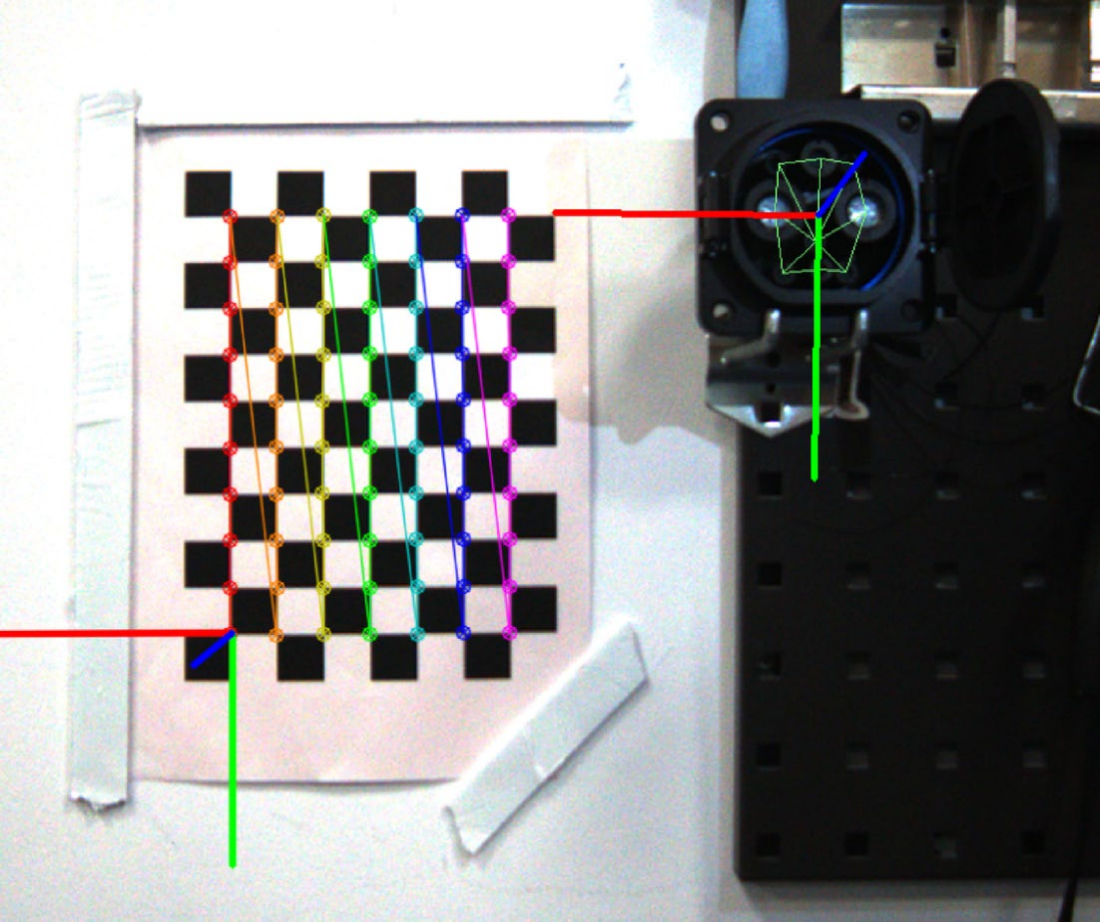
A side-by-side comparison showcases the ground truth obtained through the use of a chessboard on the left and the estimation generated by our proposed algorithm on the right.

2 **Annotations and Ground Truth**: Labelme^42^ was employed for the manual annotation of key points on the charging port, ranging from ${P_1}$ to ${P_9}$, following the sequence illustrated in Fig. 5. To precisely determine the charging port’s coordinates, a custom calibration device featuring a checkerboard and a standard EV charging port was used, as shown in Fig. 8. The charging port was securely mounted on this device to maintain a fixed pose and position relative to the calibration setup, hence enabling an indirect yet precise estimation of its real-world location and orientation. A labeled example is presented in Fig. 10.
  The methodology commenced with calibrating the camera’s intrinsic parameters, followed by utilizing a homemade calibration tool to determine its pose with respect to a checkerboard. The fixed spatial relationship between the checkerboard and the charging port facilitated the derivation of the camera’s orientation concerning the charging port. This was accomplished by calibrating the checkerboard’s pose and leveraging its known relative positions to the charging port. Figure 11 depicts a comparison between the ground truth positions obtained via checkerboard calibration and the estimated poses derived from our proposed pose extraction algorithm. In this figure, the bottom left illustrates the checkerboard’s pose, serving as a reference for gauging the charging port’s pose after transformation. The top right corner displays the poses computed by the PnP and BA algorithms, with FasterEVPoints’nine key points connected by thin green lines to highlight the resulting alignment.

**Fig. 12:**
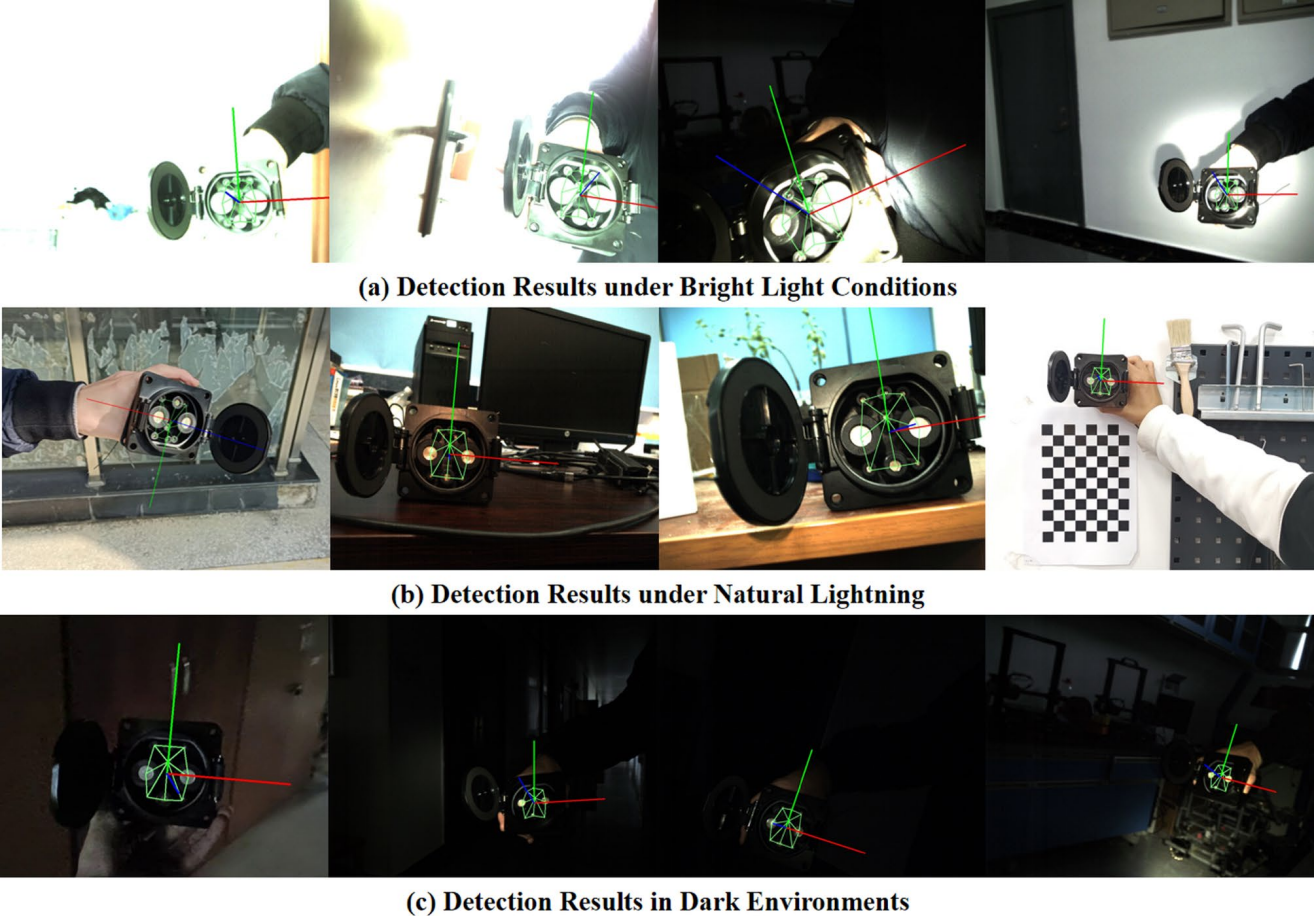
Visualization results for pose detection under diverse lighting conditions, ranging from glare, outdoor natural light, to indoor low-level lighting.

### Pose detection and estimation result

1. **Performance Estimation of Keypoints Detection**: The FasterEVPoints model was trained on our proprietary dataset using RTX 3060 GPU, with the process concluding at the 200th epoch. Stochastic gradient descent (SGD) was adopted as the optimizer, while data augmentation techniques—including random rotations, flipping, cropping, and mixup—were applied to enrich the dataset. The FasterEVPoints model demonstrated strong performance, achieving an accuracy of 0.95 and a recall of 0.96 on the validation set.
  **Fig. 13:**
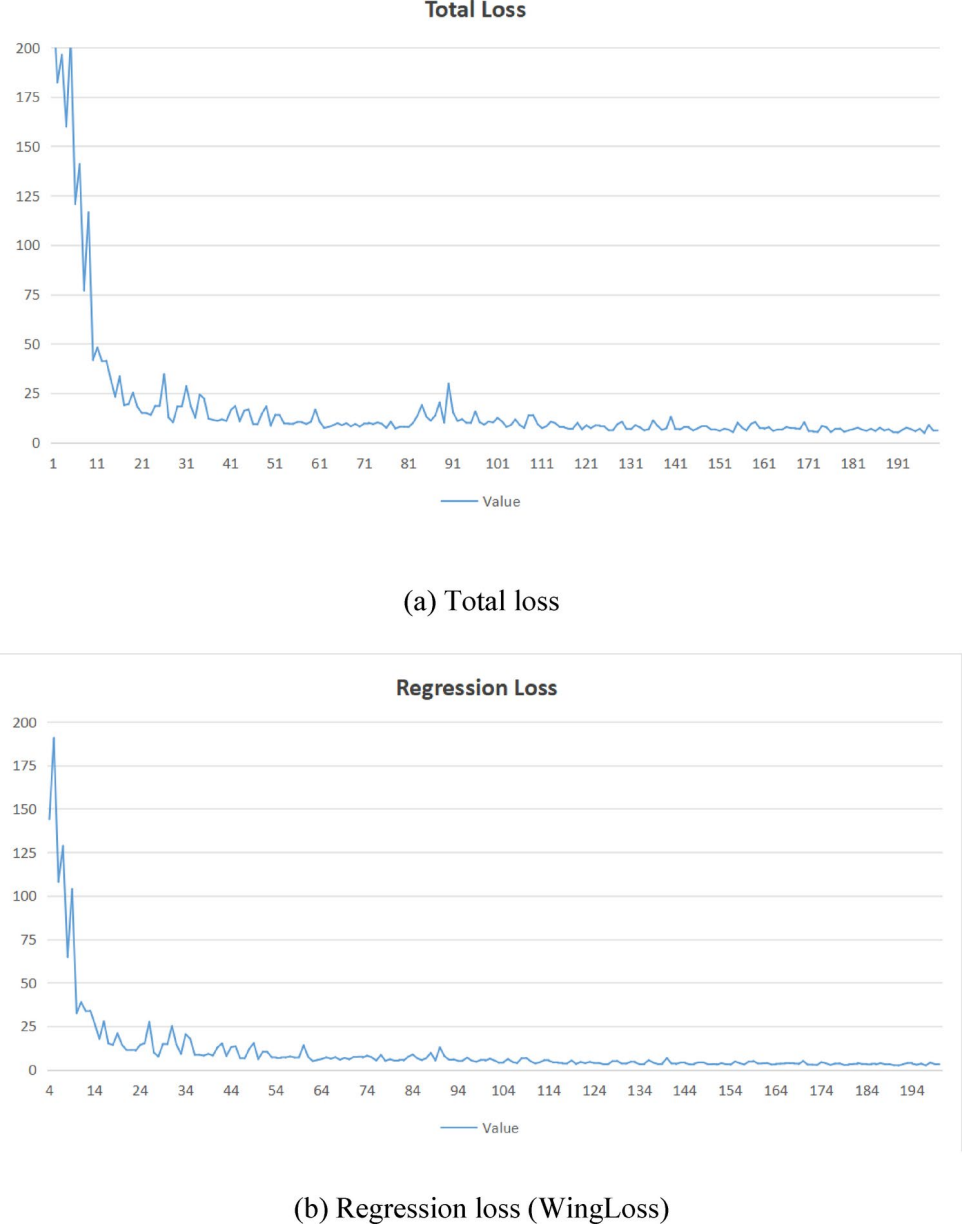
Total loss and regression loss (WingLoss) distribution of FasterEVPoints during the training. (**a**) Total loss. (**b**) Regression loss (WingLoss)
  Testing was conducted on an RTX 3060 laptop, yielding an average inference time of no more than 3ms. Representative visualizations of these results are displayed in Fig. 12. The first row of images presents outcomes under intense illumination, the second row under ambient natural light, and the third row under low-light conditions.
  Figure 13 illustrates the progression of total loss and regression loss (WingLoss) throughout training, where both curves exhibit a clear downward trend. Notably, the regression loss stabilizes around the 35th epoch, albeit at a slightly slower rate compared to the total loss, which converges by the 25th epoch. The fluctuations observed in the curves are attributed to the uneven distribution of data across epochs, affecting the rate at which the losses stabilize.

2 **Performance Estimation of Camera Pose Correction**: The methodology applies checkerboard calibration to indirectly ascertain the true values of pose, inherently introducing a margin of error. Specifically, at distances below 50 cm, the calibration error becomes pronounced, prompting the exclusion of the 20–30 cm range from the experiments.
  In pose estimation, positional error predominantly occurs along the Z direction (depth direction). For instance, at a distance of 50 cm, the error margin is approximately 6.0%, decreasing slightly to 5.2% at 75 cm. Regarding attitude estimation, errors are most prominent in the Roll direction, with the Z axis as the rotation axis. At 50 cm, the estimation error is around 4.59 degrees, which increases to about 6.16 degrees at 75 cm. Detailed error metrics are demonstrate in Table 2.
  **Table 2:** The position errors in the X, Y, and Z directions at different test distances.

Distance(cm)	X (cm)	Y(cm)	Z(cm)	Roll (°)	Pitch (°)	Yaw (°)
50	0.85	0.54	3.01	4.59	3.92	1.68
75	1.01	1.31	3.90	6.16	5.83	2.05
  **Table 3:** The results of ablation study on BA optimization on the position and orientation error.

Method	X (cm)	Y(cm)	Z(cm)	Roll (°)	Pitch (°)	Yaw (°)
No BA	1.21	1.27	4.11	8.06	7.01	2.07
BA	1.01	1.34	3.90	6.16	5.83	2.05
  An ablation study was performed to evaluate the impact of BA optimization on pose estimation accuracy, with experiments conducted at a distance of 75 cm. The results are documented in Table 3. In terms of positional accuracy, while BA optimization results in a slight increase in error along the Y direction by approximately 5.5%, it significantly reduces errors along the X and Z directions. This leads to an overall reduction in the final positional error by about 10%. For attitude accuracy, BA optimization notably decreases errors across the Roll, Pitch, and Yaw directions, with a maximum error reduction of approximately 23.6% in a single direction. Hence, BA optimization is considered essential for enhancing pose estimation outcomes.
  **Fig. 14:**
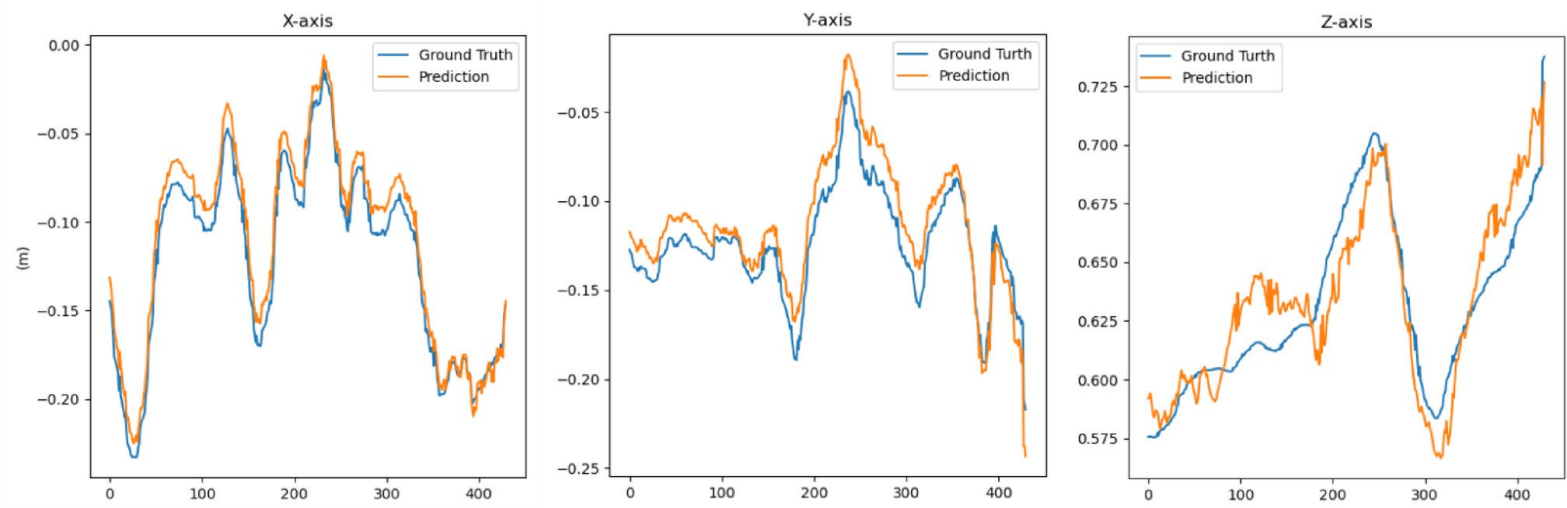
Comparison of position information obtained by our algorithm (orange lines) with reference position information from chessboard calibration (blue lines) in the same video.
  Furthermore, the algorithm was applied to video streams to validate its accuracy by comparing the positions estimated by the algorithm against the ground truth positions obtained from checkerboard calibration, as depicted in Fig. 14. The comparison was presented by overlaying the algorithm-determined positions (orange lines) with the ground truth positions (blue lines). In this analysis, algorithm’s position estimates closely align with the reference positions from checkerboard calibration along the x and y axes, revealing near-identity. However, significant fluctuations were observed along the z-axis, indicating a greater challenge in accurately estimating vertical distances compared to the horizontal directions. This underscores the varying levels of difficulty in pose estimation across different spatial dimensions.

### Model performance comparison

In this paper, two sets of comparative experiments were conducted to comprehensively evaluate the performance of FasterEVPoints. The first set focused on the keypoint localization accuracy, wherein FasterEVPoints was directly compared with YOLOv8-Pose on the electric vehicle charging port dataset. Both methods utilized the EPnP algorithm and BA algorithm for pose extraction to ensure consistency in experimental conditions.

**Fig. 15:**
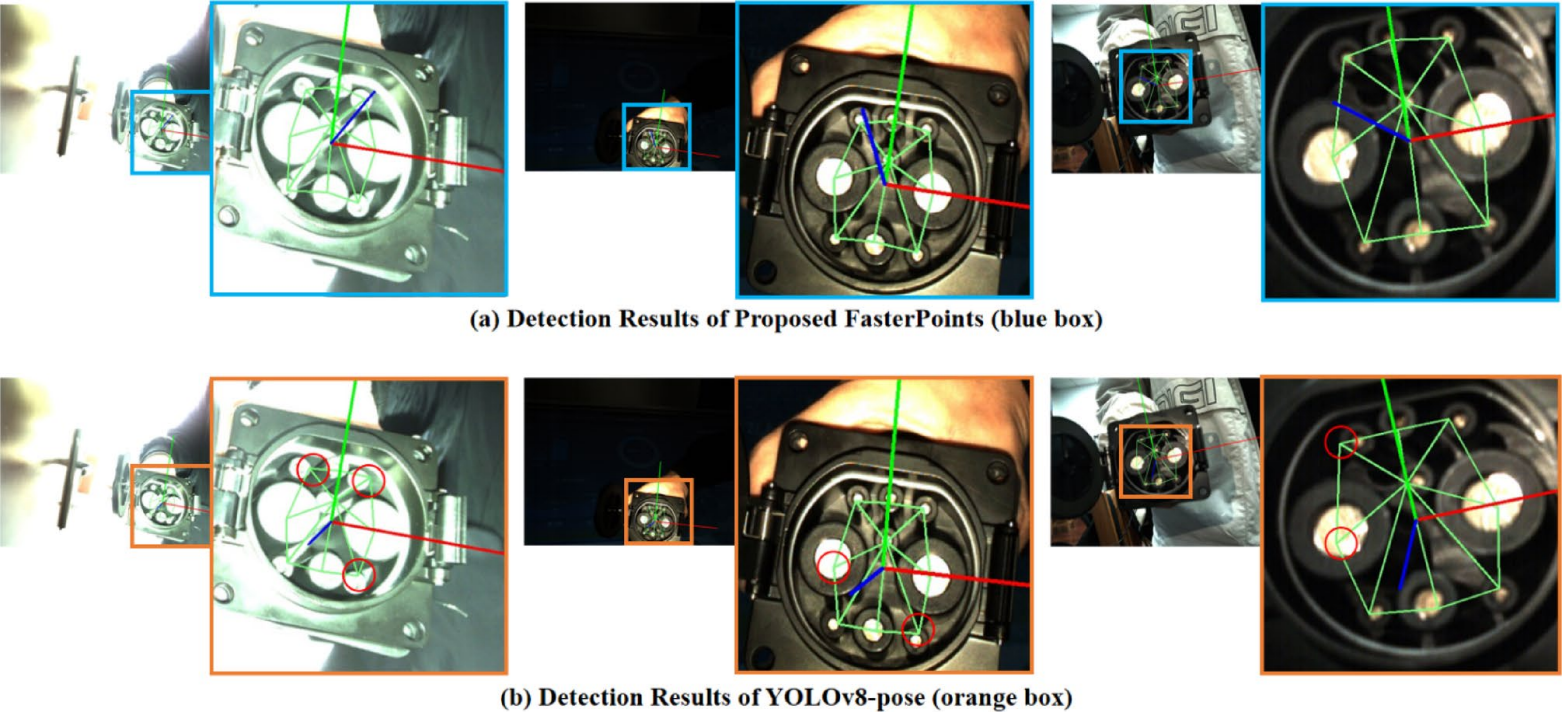
Comparison results of keypoint localization between FasterEVPoints and YOLOv8-Pose.

The results of the first group of comparative experiments are visually presented in Fig. 15. The first row of Fig. 15 clearly showcases the recognition results of the FasterEVPoints model, while the second row displays the recognition outcomes of YOLOv8-Pose. A detailed examination reveals that YOLOv8-Pose exhibits larger errors in keypoint localization relative to FasterEVPoints, particularly for the keypoints marked with red circles, where substantial deviations are evident.

**Fig. 16:**
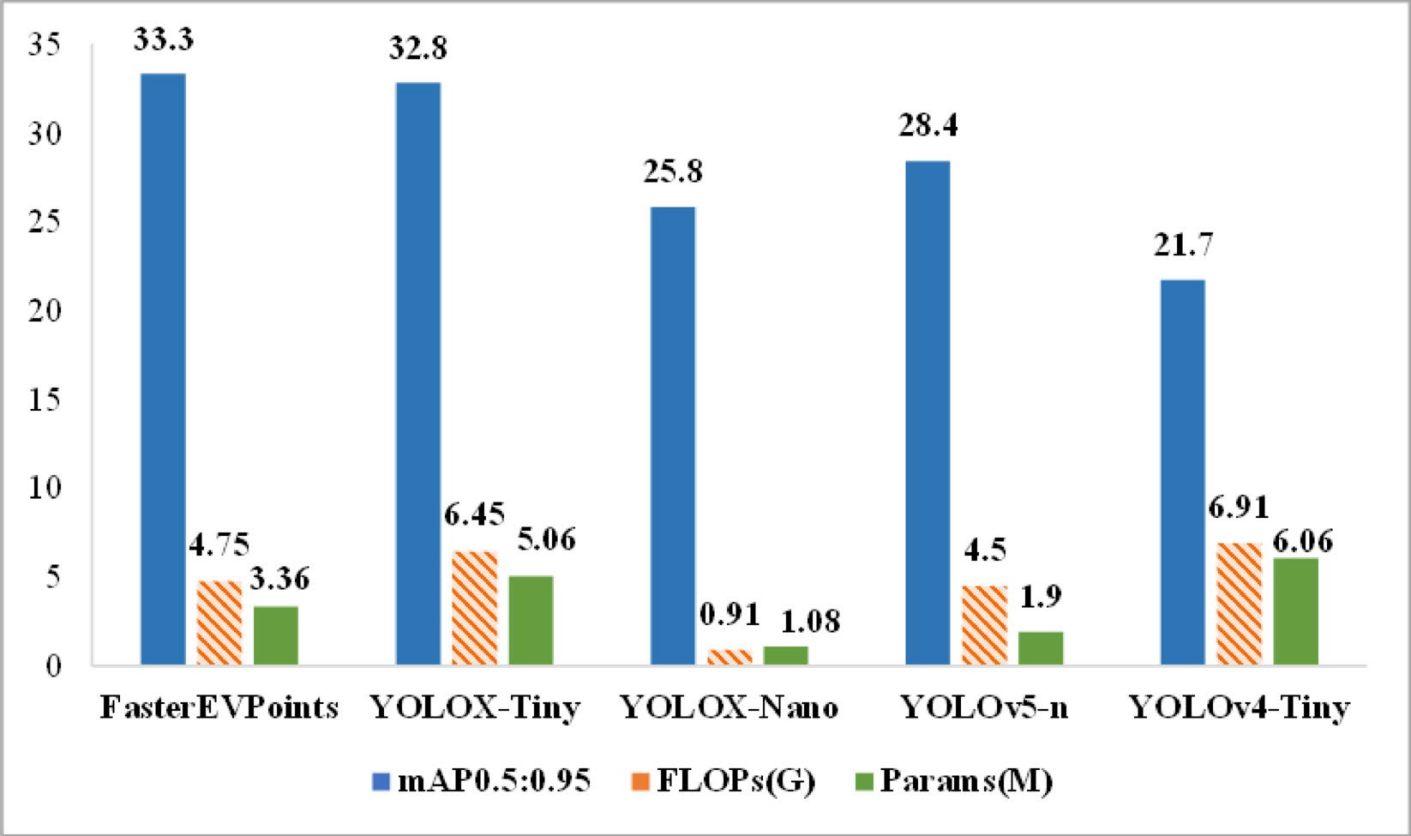
Performance comparison on the widely recognized COCO 2017 object detection task.

**Table 4:** Performance comparison between our proposed FasterEVPoints and mainstream CNN models on the widely recognized COCO 2017 object detection task.

Model	Resolution	mAP0.5:0.95	FLOPs	Params
FasterEVPoints	480*480	33.3	4.75G	3.36 M
YOLOX-Tiny^29^	416*416	32.8	6.45G	5.06 M
YOLOX-Nano ^29^	416*416	25.8	0.91G	1.08 M
YOLOv5-n ^28^	416*416	28.4	4.5G	1.9 M
YOLOv4-Tiny ^27^	416*416	21.7	6.91G	6.06 M

Since a detection bounding box can be represented by two vertices, the top-left and bottom-right corners, it can be considered as a target with two keypoints. Consequently, the proposed FasterEVPoints model is also applicable to object detection tasks. To validate the performance of our network architecture on such tasks, a second set of experiments was conducted, focusing on the MS-COCO object detection challenge. In this experiment, FasterEVPoints was integrated into the YOLO X (YOLOX )^29^ framework, adopting its loss functions and adapting the keypoints prediction mechanism to align with bounding box predictions. This approach enabled training on the widely recognized COCO 2017 object detection dataset, employing the same training methodologies and loss functions—specifically, the generalized intersection over union (GIoU) loss^43^—as used by YOLOX-Tiny. The comparative results, as summarized in Table 4 and illustrated in Fig. 16, indicate that under identical training conditions and datasets, the model outperforms YOLOX in key metrics, while also reflecting superior efficiency in terms of parameter utilization and computational resources.

Furthermore, when compared with models that incorporate more recent object detection loss functions and more advanced training techniques, FasterEVPoints maintains its competitive performance. This resilience underscores the model’s robustness, as well as the effectiveness of its underlying architecture and optimization strategies, showcasing its potential as a leading solution in the domain of object detection.

## Conclusions

This paper introduces an efficient, highly robust, and precise algorithm for the identification and localization of EV charging ports. The algorithm consists of two primary modules: a keypoint detection module and a pose correction module. Within the detection framework, we proposed the FasterEVPoints model, an advanced CNN designed specifically for keypoint detection, with a particular focus on EV charging ports. For pose correction, the initial pose is estimated using the EPnP algorithm, followed by refinement through the BA optimization algorithm, incorporating a strategic matching approach.

Leveraging a custom dataset encompassing a diverse range of lighting conditions, backgrounds, and viewing angles, the FasterEVPoints model undergoes rigorous training and evaluation, achieving an accuracy rate of 96%. Results from pose detection and estimation underline the model’s precision in keypoints identification and illustrate minimal error rates in both positional and attitudinal estimates across various distances, emphasizing the pivotal role of BA optimization in refining pose estimation outcomes. Moreover, when compared to baseline models such as YOLOX-Tiny, YOLOX-Nano, YOLOv4-Tiny, and YOLOv5-n on the MS-COCO 2017 object detection task, our model distinguishes itself by minimizing computational demands while sustaining high levels of accuracy.

Despite the algorithm’s robust performance, the study acknowledges potential inaccuracies, primarily arising from the inherent complexity and variability in the physical characteristics of charging ports. Additionally, the dataset currently does not fully encompass more extreme environmental conditions, such as heavy rain or sand-dust weather, nor does it cover a wider variety of charging ports from various electric vehicle brands and models. These limitations may lead to challenges like reduced visibility, occlusions, and noise interference, which could impact the robustness of the model. Future research endeavours are directed towards improving the algorithm’s adaptability and accuracy, particularly by expanding the training dataset to include such adverse conditions and a broader array of charging port configurations. Furthermore, the integration of more advanced machine learning techniques will be explored to refine the algorithm’s accuracy and robustness. This expansion will allow for a more thorough evaluation and improvement of the model’s performance in dynamic real-world environments.

## Data Availability

All the data used in this study can be obtained from the corresponding author upon a reasonable request (e.mail: xxywyj@sina.com ).
